# Improving Therapeutic Vaccination against Hepatitis B—Insights from Preclinical Models of Immune Therapy against Persistent Hepatitis B Virus Infection

**DOI:** 10.3390/vaccines9111333

**Published:** 2021-11-16

**Authors:** Percy A. Knolle, Li-Rung Huang, Anna Kosinska, Dirk Wohlleber, Ulrike Protzer

**Affiliations:** 1 Institute of Molecular Immunology and Experimental Oncology, School of Medicine, Technical University of Munich, 81675 Munich, Germany; dirk.wohlleber@tum.de; 2 German Center for infection Research (DZIF), Munich Site, 81675 Munich, Germany; protzer@tum.de; 3 Institute of Molecular and Genomic Medicine, National Health Research Institutes, Zhunan Town, Miaoli City 350, Taiwan; lrhuang@nhri.edu.tw; 4 Institute of Virology, School of Medicine, Technical University of Munich, 81675 Munich, Germany; anna.kosinska@helmholtz-muenchen.de

**Keywords:** hepatitis B virus (HBV), vaccination, therapeutic vaccination

## Abstract

Chronic hepatitis B affects more than 250 million individuals worldwide, putting them at risk of developing liver cirrhosis and liver cancer. While antiviral immune responses are key to eliminating hepatitis B virus (HBV) infections, insufficient antiviral immunity characterized by failure to eliminate HBV-infected hepatocytes is associated with chronic hepatitis B. Prophylactic vaccination against hepatitis B successfully established protective immunity against infection with the hepatitis B virus and has been instrumental in controlling hepatitis B. However, prophylactic vaccination schemes have not been successful in mounting protective immunity to eliminate HBV infections in patients with chronic hepatitis B. Here, we discuss the current knowledge on the development and efficacy of therapeutic vaccination strategies against chronic hepatitis B with particular emphasis on the pathogenetic understanding of dysfunctional anti-viral immunity. We explore the development of additional immune stimulation measures within tissues, in particular activation of immunogenic myeloid cell populations, and their use for combination with therapeutic vaccination strategies to improve the efficacy of therapeutic vaccination against chronic hepatitis B.

## 1. The Challenge of Chronic Viral Hepatitis

Hepatitis B Virus (HBV) infection affects almost one-third of the world’s population, and in most cases, is cleared by host anti-viral immunity [1,2]. However, more than 250 million individuals suffer from chronic hepatitis B [1], which puts them in danger of developing liver cirrhosis and liver cancer. Dysfunctional anti-viral immunity is considered the cause of persistent viral infection with virus-specific effector immune cells lacking the capacity to eliminate HBV infected hepatocytes, which is characterized by failure to achieve seroconversion to anti-HBs and the establishment of broad and strong HBV-specific T cell response [2,3]. Nevertheless, liver damage during acute and chronic hepatitis B is caused by the host´s immune response against HBV [2,4]. This suggests that a delicate balance exists between mechanisms promoting persistent infection of hepatocytes with HBV and the host´s HBV-specific immune response. Along this line, spontaneous clearance of persistent HBV infection is observed in some patients with chronic hepatitis B [5], which supports the notion that persistent HBV infection and chronic hepatitis B may be therapeutically targeted by strengthening HBV-specific immunity. 

Currently, however, efficient direct antiviral therapies using nucleoside inhibitors are used for treatment in patients with chronic hepatitis B, which inhibit HBV replication but fail to induce protective HBV-specific immunity. The main reason for direct antiviral therapies to fail to achieve a cure from chronic hepatitis B, is the establishment of a persistent form in HBV-infected hepatocytes, the so-called covalently closed circular DNA (cccDNA) that serves as an extrachromosomal template for viral replication [6,7]. Despite successful control of HBV replication by direct antiviral drugs, treatment interruption is accompanied by re-activating cccDNA and initiation of viral replication, leading again to chronic viral hepatitis. In contrast, chronic hepatitis C is successfully treated by direct acting antiviral agents [8], but this sensitivity towards antiviral therapy is based on the strict requirement of the hepatitis C virus, as an RNA virus, to continuously replicate [9]. This is not the case for HBV, which can persist via its cccDNA without replicating at all. However, HBV cccDNA is sensitive to the anti-viral activity of cytokines, such as interferons and lymphotoxin [10], but these mediators fail to eliminate all HBV cccDNA from infected hepatocytes for reasons that remain to be discovered [6,11,12]. Current direct antiviral treatment options were recently addressed in expert reviews [13,14,15].

The only way to achieve control of persistent HBV infection and cure patients from chronic hepatitis B is to eliminate HBV-infected hepatocytes or at least eradicate the HBV cccDNA pool from the liver. Thus, an urgent medical need exists to develop novel immune therapies to strengthen HBV-specific effector responses in order to cure patients with chronic hepatitis B from the virus. A successful therapeutic vaccination against HBV would also provide a cure from infection with the more pathogenic hepatitis delta virus, which requires HBV coinfection to replicate, and against which few therapeutic options exist [16]. Furthermore, therapeutic vaccinations could prevent the occurrence of the sincere sequelae of continuous immune-mediated liver damage during chronic viral hepatitis that can result in liver cirrhosis and liver cancer. 

## 2. Immunopathogenesis of HBV Infection and Chronic Viral Hepatitis

**Figure 1:**
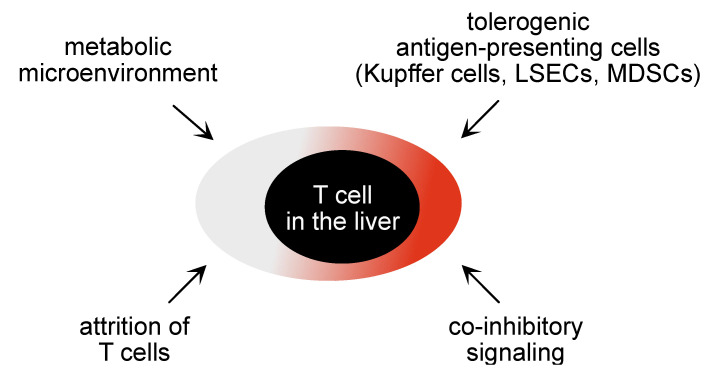
Schematic illustration of factors locally influencing T cell function in the liver.

Understanding the immunopathogenesis of chronic hepatitis B is key for a rationale development of novel immune-based therapies. HBV is a strictly hepatotropic virus that selective targets hepatocytes and selectively replicates within hepatocytes [2]. This strict hepatotropism of HBV is most likely one of the reasons why mounting of protective immunity poses particular challenges for the host´s immune response. Successful immunity against HBV infection is characterized by induction of a strong CD4 and CD8 T cell response, specific for many different viral epitopes and presence of effector CD8 T cells, as well as induction of B cell immunity against HBV that is characterized by neutralizing antibodies against HBV surface antigens [17,18,19]. In contrast, development of a persistent HBV infection is associated with a dysfunctional immune response against HBV [2,20]. Several factors have been associated with induction of persistent HBV infection (see Figure 1).

First, HBV infection fails to elicit strong innate immunity and inflammation, which is necessary for maturation of antigen-presenting cells to induce protective immunity and for virus-specific immune effector cell populations to selectively localize to the site of infection. Lacking pattern recognition and lacking induction of cell-intrinsic immunity by HBV has been recognized as a major obstacle in raising anti-viral immunity [21,22,23,24], since inflammation is required for functional maturation of antigen presenting cells to mount protective immunity [25]. Activation of pattern-recognition pathways and induction of an inflammatory environment is therefore likely to play an important role in the generation of strong antiviral immunity in the liver. 

Second, the restriction of HBV replication and gene expression to hepatocytes requires cells that use endocytosis for antigen acquisition and present HBV antigens on MHC molecules to virus-specific T cells. While antigen-uptake via receptor-mediated endocytosis is well established for induction of MHC-II restricted CD4 T cell immunity, presentation of endocytosed antigens on MHC-I molecules to CD8 T cells requires special competence of the antigen-presenting cell for a process called cross-presentation [26]. Thus, only certain professional antigen-presenting cells, such as functionally matured monocytes, can execute this cross-presentation of antigens released from virus-infected hepatocytes [27]. Furthermore, a complex interaction between different immune cell populations in distinct micro-anatomic niches within lymphoid tissues is required to generate antigen-specific CD8 T cells through cross-presenting dendritic cells [28]. Overall, this is believed to cause a failure to properly prime HBV-specific immunity, which then results in a dysfunctional HBV-specific immune response.

Third, the liver microenvironment is known for its tolerogenic function and contributes to down-tuning of effector T cell responses in the liver [29]. Liver-resident tolerogenic antigen presenting cells, such as liver dendritic cells and liver sinusoidal endothelial cells (LSECs), render CD4 and CD8 T cells dysfunctional, thereby attenuating anti-viral T cell immunity locally in the liver [30,31,32,33]. Antigen-presentation by hepatocytes themselves lead to clonal elimination of antigen-specific T cells and may thereby contribute to the attrition of T cell responses [34,35]. Hepatic stellate cells engage in veto function preventing local activation of specific T cells through professional antigen-presenting cells in the liver, and liver macrophages may further contribute to development of T cell dysfunction [36,37].

Fourth, regulatory immune cell populations in the liver such as regulatory T cells, but also myeloid-cell derived suppressor cells (MDSCs) are present in the liver microenvironment and contribute to local inhibition of T cell immunity [38,39,40,41]. Fifth, the liver micromilieu is particularly rich in regulatory mediators, such as IL-10 or TGF-β, derived from local immune cell populations in the liver, such as Kupffer cells, dendritic cells, or hepatic stellate cells, and may contribute to local skewing of virus-specific immune effector functions [42,43,44]. 

Fifth, the continuous exposure to antigen appears to be a key driver of T cell dysfunction. For experimental viral infections, such as lymphocytic choriomeningitis virus infection, the mechanisms mediating this dysfunction of virus-specific T cells have been described as a state of exhaustion that is determined by the exhaustion promoting transcription factor TOX [45,46,47,48]. In chronic hepatitis B, virus-specific T cells are also dysfunctional, but the mechanisms determining their dysfunction remain to be discovered. Recently it was found that HBV-specific T cells in chronic hepatitis suffer from metabolic disturbances that can affect their effector functions [49,50]. Thus, cell-intrinsic regulation of effector function of virus-specific T cells may also contribute to the lack of immune control of HBV infection.

Thus, a large number of immune inhibitory mechanisms operate locally in the liver to control immune effector cell functions and will have to be taken into account when developing novel immune therapies that aim to increase immune effector functions in the liver. Moreover, target cell killing in the liver also seems to be subject to regulation by target cells themselves. Expression of antigens at low levels on MHC-I molecules by hepatocytes and a lack of MHC-II on hepatocytes unless there is significant inflammation protects them from effector cell killing [51], and may thus establish a further level of T cell dysfunction in the liver. Finally, continuous exposure towards antigens expressed in the liver for prolonged periods of time is associated with development of immune tolerance, which includes generation of regulatory immune cell populations [52]. Taken together, numerous mechanisms impede generation as well as execution of virus-specific effector T cells.

Beyond alterations in T cell immunity, there are also contributions of HBV itself to persistence of infection. As already mentioned, the establishment of the extrachromosomal persistence form, the covalently, closed circular HBV DNA is associated with viral persistence. HBV cccDNA is extraordinarily stable and may serve as template for viral gene expression and initiate a virus rebound even long time after the active HBV replication has ceased [6]. It remains an open question whether a shut-down of HBV gene expression upon cytokine exposure may help infected hepatocytes to escape from killing by virus-specific effector T cells [53]. Presentation of antigens on MHC I molecules is typically related to ongoing gene expression and processing of defective ribosomal products for presentation on MCH-I molecules [54,55], so the consequences of stalling HBV gene expression for subsequent recognition by virus-specific effector T cells remains unclear. Furthermore, under immune pressure HBsAg-escape mutations develop, that can contribute to the failure of immune control against HBV infection even after vaccination [56]. Since depletion of B cells by anti-CD20 therapy leads to reactivation of HBV infection [57], continuous virus control by virus-specific B cells appears to be an important part of immune control of HBV infection. However, recent studies identified broadly neutralizing antibodies that can overcome these escape mutants and provide protection [58].

On the other hand, there is a large amount of viral antigens expressed in hepatocytes upon active viral replication. Recent studies indicate that expression of these viral antigens in the liver rather that secretion of viral antigens and presentation on non-hepatic antigen presenting cells induces antigen-specific immune tolerance [59,60].

Finally, mutated viral proteins may contribute to a viral immune escape if T cell recognition of infected hepatocytes is impaired. Although HBV is a DNA virus, it replicates via reverse transcription allowing mutations in the viral genome. Due to the very compact viral genome with largely overlapping open reading frames, however, most of the resulting variants are defective and immune escape variants remain rare.

Thus, a combination of factors influences the immune response to infection with HBV, generation of virus-specific effector T cells and elimination of HBV-infected hepatocytes. It is worth noting that clearance of HBV in a natural host, i.e., chimpanzees, requires several months [61,62], which is clearly distinct from the immune response to other viruses like influenza targeting lung tissue where rapid immune responses are observed [63]. This requirement for a prolonged time period to clear infected hepatocytes from the liver not only after HBV, but also after HAV or HCV infection points towards particular obstacles that have to be overcome by the host´s immune response, to mount virus-specific immunity and eliminate virus-infected hepatocytes. 

## 3. Strategies for Therapeutic Vaccination against Chronic Hepatitis B

Different approaches have been used to establish a therapeutic vaccination against chronic hepatitis B. These were most often based on novel insights into the immunopathogenesis of HBV infection and novel technologies to improve the strengths of virus-specific immunity. However, one of the major problems in developing immune therapies against chronic hepatitis B is the lack of a suitable animal model that faithfully reflects all features of HBV infection in humans [64]. Human HBV shows strict species restriction. Only chimpanzees are susceptible for HBV infection, and important discoveries were made on HBV infectiousness and anti-viral immune responses in this model [4,62,65], before research was stopped for ethical reasons. While infection models exist for individual animal species with their particular hepatitis B viruses, such as, e.g., the duck and duck hepatitis B virus (DHBV), the woodchuck and woodchuck hepatitis B virus (WHBV), these models are restricted by important differences between the viruses and human HBV, with antigen being non-compatible as well as marked differences in immune responses and a lack of tools to study virus-specific immunity. Mice, as preferred preclinical animal models to study immune pathogenesis, are also employed for the study of HBV pathogenesis. However, to deliver HBV into hepatocytes in a species were infection is not possible, different strategies have been developed: first, genetic manipulation (transgenic mice expressing the HBV genome); second, hydrodynamic injection of HBV genomes or third, viral carriers for delivery of HBV genomes into hepatocytes [66,67]. Thus, most of our knowledge on the immunopathogenesis in persistent HBV infection and experimental approaches targeting particular immune mechanisms to control persistent infection have been generated in non-optimal models of HBV infection. 

Numerous clinical trials have been performed in patients with chronic hepatitis B to explore the importance of particular concepts how to re-install protective immunity once persistent HBV infection had established [68,69,70]. In prophylactic vaccines, an emphasis is on the induction of immunity against the surface antigens of HBV in order to elicit neutralizing anti-HBs antibodies and prevent infection. The induction of HBs-specific CD8 T cells that target and eliminate HBsAg-expressing infected hepatocytes is less important. In contrast, in therapeutic vaccination also other viral antigens, in particular, HBcore antigen and the viral polymerase are targeted to increase the breadth of the virus-specific effector T cell response and a focus is on the induction of potent CD4 and CD8 T cell responses. In general, all strategies for developing therapeutic vaccination against chronic hepatitis B included a lowering of viral replication.

In the following, we will review the different strategies used for development of therapeutic vaccination for chronic hepatitis B and their outcome.

## 4. Strengthening the Immunogenicity of Vaccination against Chronic Hepatitis B

The conceptual idea behind the strategy for therapeutic vaccination lies in the assumption that a defective induction of HBV-specific B and T cell immunity is responsible for the lack of virus clearance [69,71,72,73]. Numerous approaches have been taken to increase the immunogenicity of vaccines against chronic hepatitis B, and thereby mount strong virus-specific immunity against the surface, nucleocapsid, or polymerase antigens of HBV that should then control HBV infection by induction of virus-specific neutralizing antibodies and elimination of virus-infected hepatocytes through effector T cells. The first attempts to establish therapeutic vaccination against chronic hepatitis B in patients were undertaken by increasing the number of administrations of vaccines, which were originally developed for use as prophylactic vaccines and, therefore, targeted HBsAg. Most vaccines contain alum as adjuvant, which has been shown to involve induction of innate immunity through still rather ill-defined pathways [74] and induces a strong Th2 bias. In an attempt to increase immunogenicity, prophylactic vaccines were injected at different sites and in particular intradermally, because local intradermal activation of immune responses is considered to be superior [75]. In addition, T cell-targeted vaccines or combinations of HBsAg and HBcAg as immunogens were investigated for their efficacy of therapeutic vaccination [76,77,78]. However, all these approaches failed to achieve a cure in patients with chronic hepatitis B [59,70,71]. 

The key for the success of prophylactic recombinant vaccines is the use of adjuvants [74] that are the basis for providing signal 3 to antigen presenting cells and induction of local inflammation and, therefore, properly prime T cell immunity. Hereby alum, by inducing a strong Th2 bias, prevents the induction of effector T cell responses. Using other adjuvants, in combination with particulate HBV antigens, have shown promising results at least in preclinical models [79]. The discovery of ligands for immune sensory molecules, such as ligands for TLR7, TLR8, TLR9, and cyclic-di-AMP as a ligand for the cGAS/STING pathway, as well as ligands for the cytosolic RNA-recognition receptor RIG-I or MDA-5, triggered substantial interest in their therapeutic use for chronic hepatitis B. Adjuvants serve the purpose of triggering inflammation and, more specifically, functional maturation of dendritic cells, thereby increasing the strength of the immune response against recombinant antigens. For instance, TLR9 is expressed on professional antigen-presenting B and dendritic cells, and ligands of TLR9 used as an adjuvant may therefore have a positive effect on the immunogenicity against antigens included in a vaccine [74,80]. Recently, a new prophylactic vaccine against hepatitis B was brought to the market that includes a TLR9-ligand as adjuvant showing superiority to alum-based vaccines [81]. It will be interesting to see whether it will show efficacy in a therapeutic setting against chronic hepatitis B.

Given the constant exposure of the persistently infected host to HBV antigens, in particular high levels of circulating HBsAg, it was reasoned that application of adjuvants might suffice to trigger HBV-specific immunity [74]. Along this line, oral delivery of TLR-ligands, considered to lead through the portal venous drainage of the gut to delivery of TLR-ligands to the liver, was evaluated as a treatment option for chronic hepatitis B [82,83]. Moreover, ligands for cytosolic immune sensory receptors, such as for the helicase RIG-I, were shown to be effective in controlling experimental HBV infection [84,85,86]. In clinical trials, neither control of HBV nor cure from chronic hepatitis B has been achieved using TLR agonists so far, indicating that the application of a TLR agonist may not result in induction of HBV-specific immunity, and triggering innate immunity and inflammation alone may not be sufficient to overcome immune tolerance and achieve control of chronic hepatitis B. However, alternative pattern-recognition receptor agonists triggering TLR8, Rig-I, or STING are currently evaluated in clinical trials; it will be interesting to see the outcome. 

The choice of the immunogen in a vaccine is also of key importance. Whereas prophylactic vaccines only need to elicit neutralizing antibodies directed against the HBV envelop proteins, therapeutic vaccines most likely need to induce a broad T cell response and, thus, should include other HBV antigens, such as HBV core and polymerase [70]. An interesting approach identified the HBV X protein as a valuable target for vaccinations using a preclinical model of persistent HBV infection [87]. The HBV X protein is expressed at much lower levels than other viral proteins and its low abundance in the infected liver may provide a better target for a vaccination, since high antigen expression levels of model viruses are often associated with development of T cell exhaustion [88]. However, hepatocytes with their low-level MHC-I expression may also fail to present any peptide from this small X protein.

A further approach to increase immunogenicity of vaccines in the setting of chronic hepatitis B is the development of heterologous prime-boost vaccination strategies [70]. The combinations of adjuvanted protein-based vaccines, DNA vaccination, and vector-based immunizations have been tested in various preclinical models of persistent HBV infections, and have yielded promising results [89,90,91]. Conceptually, development of vaccines using viral vectors to deliver HBV antigens and to elicit strong anti-viral immunity provides an interesting approach for development of a therapeutic vaccine. Viral vectors employed for this purpose include adenoviral vectors (mostly non-human adenoviral vectors e.g., from chimpanzee), yellow fever virus vectors, and modified vaccinia virus Ankara (MVA)-based vectors [89,91,92]. The combination of a protein prime followed by an MVA-boost, referred to as TherVacB, has proven to be very successful in different preclinical models of persistent HBV infection [59,91,93], making it an excellent candidate for a therapeutic vaccination strategy to cure HBV. A key advantage of heterologous prime-boost vaccination is the induction of both, CD8 and CD4 T cell responses. Since CD4 T cells are instrumental for overcoming experimental chronic infection and have been shown to be associated with clearance of chronic hepatitis B in patients [18,94], the concomitant induction of anti-viral CD8 and CD4 T cell immunity may be critical for vaccine efficacy. Different combinations of prime and boost vaccinations are currently tested in clinical trials for efficacy in overcoming HBV-specific immune tolerance and control of chronic hepatitis B (Table 1). 

The necessity for induction of potent HBV-specific immunity to overcome HBV-specific tolerance in the setting of chronic hepatitis B [2,95] may be best addressed by heterologous vaccination strategies. Such heterologous prime-boost vaccination strategies have proven beneficial for increasing immunity in other viral infection, such as SARS-CoV-2 [96,97]. The ongoing clinical trials will provide us with important information on the potency of heterologous prime-boost therapeutic vaccination in patients with chronic hepatitis B.

## 5. Local Support for T Cell Immunity in the Liver to Increase Efficacy of Therapeutic Vaccination

The liver has unique functions of as tolerogenic organ [2,29,71,98], and may curtail the effector function of T cells generated by a therapeutic vaccination, once they recognize their antigen in the liver. Such a threat of reducing the efficiency of therapeutic vaccination might not be possible to address by increasing the immunogenicity of therapeutic vaccination, but may require additional measures to enable effector T cells locally in the liver to control viral replication and to eliminate virus-infected cells. Three different approaches have surfaced over the last years that have the potential to increase the efficacy of therapeutic vaccination

Combination of therapeutic vaccination with inhibition of co-inhibitory receptor signaling in T cells may be an option to increase efficacy of vaccination. Expression of PD1 was shown to be increased in virus-specific T cells during persistent infection with different viruses and blockade of PD-1 was shown to increase the effector function of HBV-specific T cells from patients with chronic hepatitis B or in preclinical models [99,100,101,102,103]. However, anti-PD-1 treatment of patients with chronic hepatitis B and hepatocellular carcinoma did not reveal an effect of checkpoint inhibition on restoration of HBV-specific immunity and consequent reduction in viral replication [104]. Notwithstanding this lack of an immunity-restoring effect of anti-PD-1 therapy, the combination of therapeutic vaccination with checkpoint inhibition may be beneficial to overcome the local tolerogenic microenvironment of the liver, where high expression levels of PD-L1 are observed [33,105]. Currently, one clinical trial explores the potential of an anti-PD-1 antibody in the context of therapeutic vaccination in chronic hepatitis B patients (Table 1). 

High-level antigen expression has been identified as a key factor in reducing the efficacy of effector T cell responses [88,106] and has been suspected to play a role in attenuating HBV-specific immunity during chronic infection [95,106,107]. Recently, we have demonstrated that reduction of HBV-replication and gene expression through an siRNA or shRNA approach before therapeutic vaccination in two different models of persistent HBV infection in mice increased the efficacy of therapeutic vaccination, to eliminate HBV-expressing hepatocytes and achieve control of persistent infection [59]. Of note, neither induction of neutralizing antibodies reducing circulating HBsAg levels nor siRNA/shRNA-mediated knockdown of HBV gene expression alone was able to restore HBV-specific immunity [59]. This strengthens the notion that local inhibition of T cell effector function in the liver adds a separate hurdle to T cells generated by therapeutic vaccination to achieve control over persistent infection.

Although the liver is known for its tolerogenic function and has the capacity to curtail T cell effector functions, strong immunity can be built in the liver against pathogens, which seems to be strongly linked to the composition of myeloid cells in the liver [108]. In particular, replacement of tolerogenic liver macrophages (Kupffer cells), through pro-inflammatory monocytes, is correlated to the induction of immunity in the liver [109]. Recently, a distinct population of Kupffer cells was identified that is capable of cross-presenting hepatocyte-derived antigens to CD8 T cells upon stimulation by IL-2 and, thereby, increase HBV-specific immunity against infected hepatocytes [110].

**Figure 2:**
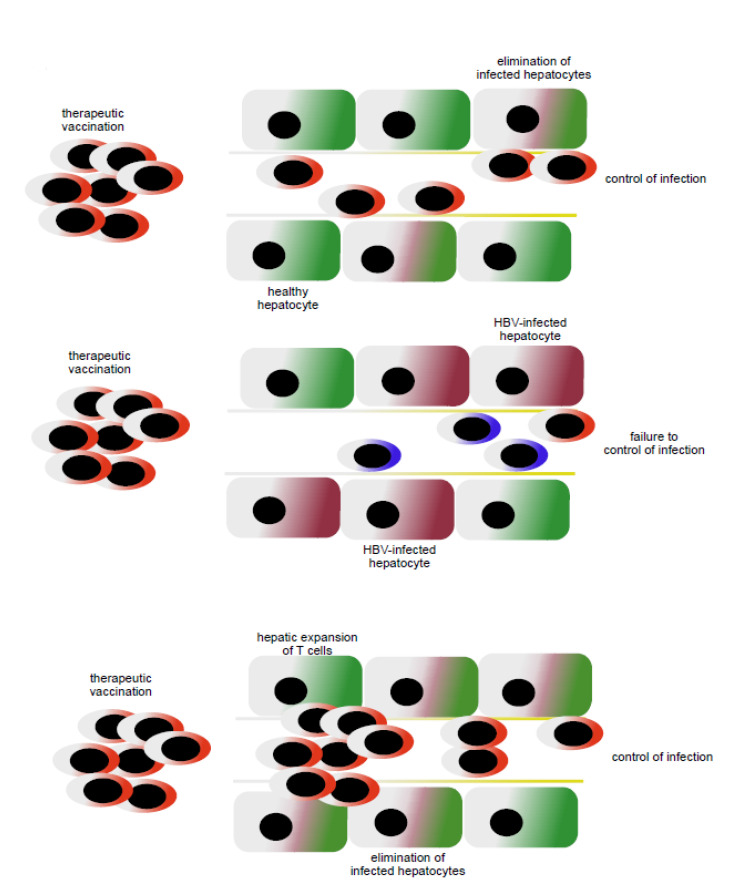
Virus-specific T cells generated by therapeutic vaccination in lymphoid tissues recognize virus-infected hepatocytes and eliminate infection if the numbers of hepatocytes are limited (**upper panel**), whereas therapeutic vac-cination fails to control viral infection if the numbers of hepatocytes are too high (**middle panel**). Combination of in-duction of virus-specific T cells in lymphoid tissue through therapeutic vaccination and local expansion of T cells in the liver acts synergistically to achieve control of infection (**bottom panel**).

Importantly, the accumulation of inflammatory monocytes in the liver as a consequence of TLR-induced inflammation leads to a massive expansion of T cells in the liver within dedicated anatomic niches termed iMATEs (intrahepatic myeloid cell aggregates associated with T cell expansion) [111]. The T cells expanding within iMATEs have potent effector potential and are capable of rapidly eliminating virus-infected hepatocytes [111]. Such TLR-induced and myeloid cell-mediated increase in effector T cell numbers in the liver also triggers elimination of hepatocytes expressing transgenes and establishes memory responses [112]. Recently, we have combined therapeutic vaccination and iMATE-induction in a model of persistent HBV infection in mice. The combination of heterologous prime-boost vaccination (HBV antigen prime vaccination followed by MVA-HBV boost vaccination) with iMATE induction leads to increased numbers of HBV-specific effector T cells in the liver [113]. Furthermore, it also improves the efficacy of therapeutic vaccination to eliminate HBV-expressing hepatocytes from the liver and clearing persistent infection [113]. This demonstrates a synergistic activity of therapeutic vaccination followed by local amplification of T cell immunity in the liver (see Figure 2). High numbers of HBV-expressing hepatocytes limit the efficacy of the heterologous prime-boost therapeutic vaccination [91]. The ability of the combination of therapeutic vaccination with iMATE-induced T cell expansion in the liver to control infection, higher levels of HBV infection than that controlled by therapeutic vaccination alone, further strengthens the notion that therapeutic vaccination to generate high numbers of virus-specific effector T cells, presumably in secondary lymphoid tissues and local expansion of T cells in the liver, are two separate mechanisms that synergize to increase the efficacy of therapeutic vaccination against virus-infected hepatocytes in the liver. 

In summary, heterologous prime-boost vaccination strategies employ synergistic principles to increase the efficacy of vaccinations against chronic viral infections. Opportunities for a further increase in vaccine efficacy may lay in the combination of local amplification of vaccine-induced immune responses, such as the above-mentioned boosting of vaccine-induced T cell immunity by increasing the strength of T cell immunity locally in the liver. Furthermore, improvement of hepatic targeting and delivery strategies for molecules boosting T cell immunity in the liver may provide further benefits for overcoming immune tolerance during chronic inflammation. 

## Figures and Tables

**Table 1:** List of current clinical trials investigating heterologous prime boost therapeutic vaccines against chronic hepatitis B.

Vaccine Candidates	Components	Stage	Reference
GSK3528869A	ChAd155-hIi-HBV HBc-HBs/AS01B-4 MVA-HBV	Phase 1	NCT03866187
VTP-300	ChAdOx1-HBV MVA-HBV Nivolumab	Phase 1/2	NCT04778904
TherVacB	HBs and HBcore antigen MVA-HBV	Phase 1 (in prep)	Available online: https://www.thervacb.eu/ (accessed on 5 October 2021)

## Data Availability

Not applicable.
